# Preoperative staging of gastric cancer: a prospective comparison of gastric filling ultrasonography and enhanced CT with implications for surgical decision-making

**DOI:** 10.3389/fsurg.2026.1925096

**Published:** 2026-10-01

**Authors:** Xinxin Qin, Junmei Zhao, Litao Ge, Song Wu, Xiaofeng Sun, Wei Li

**Affiliations:** 1 Department of Gastrocolorectal Surgery, General Surgery Center, The First Hospital of Jilin University, Changchun, China; 2 Department of Cadre’s Wards Ultrasound Diagnostics, Ultrasound Diagnostic Center, The First Hospital of Jilin University, Changchun, China

**Keywords:** clinical staging, enhanced computed tomography, filling ultrasound, gastric cancer, ultrasound

## Abstract

**Background:**

Accurate preoperative staging is essential for optimizing treatment strategies in gastric cancer. While gastric-enhanced CT is widely used, its accuracy remains suboptimal, particularly for early-stage tumors. Gastric filling ultrasonography, a non-invasive technique using oral contrast to displace intraluminal gas, offers potential advantages in mural visualization. This prospective study compared the diagnostic performance of gastric filling ultrasonography and gastric-enhanced CT against postoperative pathological staging.

**Methods:**

We enrolled 406 patients with histologically confirmed gastric cancer who underwent both imaging modalities prior to surgery. Preoperative T and N stages assigned by each modality were compared with pathological results. Diagnostic accuracy, sensitivity, specificity, and area under the ROC curve (AUC) were calculated. McNemar's test was used for paired comparisons, DeLong test for AUC comparisons, and Kappa statistics for agreement assessment. Univariate logistic regression identified factors affecting ultrasound accuracy.

**Results:**

For T-staging, overall accuracy was 72.7% (295/406) for ultrasound vs. 63.5% (258/406) for CT (*P* < 0.001). Ultrasound significantly outperformed CT in T1 (81.5% vs. 56.3%, *P* = 0.009) and T2 (81.1% vs. 67.9%, *P* < 0.001) stages. For N-staging, accuracy was 82.0% (333/406) for ultrasound vs. 60.1% (244/406) for CT (*P* < 0.001). Kappa agreement with pathology was substantial for ultrasound T-staging (*κ* = 0.625) and N-staging (*κ* = 0.748), compared with moderate agreement for CT (*κ* = 0.508 and 0.395, respectively). In multivariate analysis, vascular infiltration was the only independent factor associated with ultrasound T-staging accuracy.

**Conclusions:**

Gastric filling ultrasonography demonstrates superior accuracy over gastric-enhanced CT for preoperative T1/T2 and N-staging, with substantial agreement with pathological findings. This non-invasive and radiation-free examination method can serve as an effective complement to CT, and is particularly suitable for gastric cancer at the T1 and T2 stages. We have proposed a complementary imaging protocol to assist in surgical decision-making, which is expected to enable clinicians to make more accurate preoperative staging assessments for patients with gastric cancer.

**Registration:**

It was registered in the ClinicalTrials.gov (NCT05596864).

## Introduction

Gastric cancer remains the fifth most common malignancy worldwide and the fourth leading cause of cancer-related mortality (1). Its etiology is multifactorial, with *Helicobacter pylori* infection representing the predominant risk factor, in addition to genetic predisposition, dietary habits, ethnicity, and lifestyle choices (2, 3). Accumulated evidence indicates that patient survival and prognosis are closely correlated with the depth of gastric wall invasion (T stage), lymph node involvement (N stage), and distant metastasis (M stage) (4). Accurate preoperative staging therefore has direct implications for treatment selection and long-term outcomes (5, 6). Early and precise staging, followed by timely surgical intervention or neoadjuvant therapy, may substantially improve both prognosis and quality of life.

Currently, preoperative evaluation of gastric cancer relies primarily on contrast-enhanced CT, magnetic resonance imaging (MRI), and endoscopic ultrasonography (7, 8). Each of these modalities, however, has inherent drawbacks. Gastric-enhanced CT entails considerable radiation exposure; MRI is costly and less widely accessible; and endoscopic ultrasound, while accurate, is invasive and poorly tolerated by some patients. Ultrasound technology has gained increasing clinical acceptance owing to its safety, affordability, and real-time imaging capability. Nevertheless, its application in gastrointestinal diseases has historically been constrained by intraluminal gas and food residue, which degrade image quality and obscure mural details. Prior to the introduction of oral contrast agents, ultrasound was considered largely ineffective for gastric evaluation by many clinicians. The advent of gastric filling ultrasonography—which involves oral administration of an echogenic contrast medium to displace intraluminal gas and contents—has substantially overcome these limitations, enabling clear visualization of the gastric wall layers.

When a gastric tumor is present, sonographic findings typically include disruption of the normal five-layer architecture and loss of mural continuity. Most published studies on gastric filling ultrasound have been retrospective in design and have often incorporated intravenous contrast enhancement (9, 10). More recently, this technique has been applied to screening and preoperative staging of early gastric cancer (9, 11). However, prospective data comparing its diagnostic performance with that of gastric-enhanced CT across all stage categories remain limited.

To address this gap, we designed a prospective study to systematically evaluate and compare the diagnostic accuracy of gastric filling ultrasonography and gastric-enhanced CT against postoperative pathological findings in a large cohort of gastric cancer patients. Furthermore, we aimed to identify specific clinical subgroups, such as those defined by tumor location or abdominal wall thickness, in which ultrasound confers the greatest incremental benefit. Ultimately, we propose a complementary imaging algorithm to assist gastrointestinal surgeons in making more rational and individualized treatment decisions.

## Methods

### Study population and data collection

This study included patients diagnosed with gastric cancer at the First Hospital of Jilin University between January 2022 and January 2024. All participants had histologically confirmed gastric adenocarcinoma based on endoscopic biopsy. Each patient underwent preoperative gastric-enhanced CT, gastric filling ultrasonography, and corresponding clinical staging at our institution. Patients were consecutively screened and enrolled between January 2022 and January 2024. Patients receiving preoperative neoadjuvant therapy or other anti-tumour treatments were excluded, as such therapies alter gastric wall and lymph-node features and would bias imaging-based T/N-staging assessment. Gastric-filling ultrasonography and enhanced-CT were both performed within 7 days before surgical resection. Patients without postoperative surgical-specimen pathological confirmation were excluded because histopathology served as the reference standard for staging evaluation. Complete postoperative histopathological data were available for all included cases. We collected demographic and clinicopathological variables including age, sex, maximum tumor diameter, histological type, grade of differentiation, pathological T and N categories, and tumor location. The diagnostic accuracies of gastric filling ultrasound and gastric-enhanced CT for preoperative staging were compared against the pathological reference standard. A flowchart illustrating the study design is presented in Figure 1. The study protocol was approved by the Ethics Committee of the First Hospital of Jilin University (approval no. 24K028-001) and was registered in the ClinicalTrials.gov (NCT05596864). The study protocol, participant information and written informed-consent documents were submitted to the Ethics Committee of The First Hospital of Jilin University on December 2021. Provisional institutional ethical authorisation to commence patient recruitment for this prospective observational study was granted before enrolment initiation in January 2022, with the formal numbered ethics-approval document (approval no. 24K028-001) subsequently issued on 18 January 2024 following internal administrative procedures. The informed-consent procedure was reviewed and approved in December 2021.

**Figure 1:**
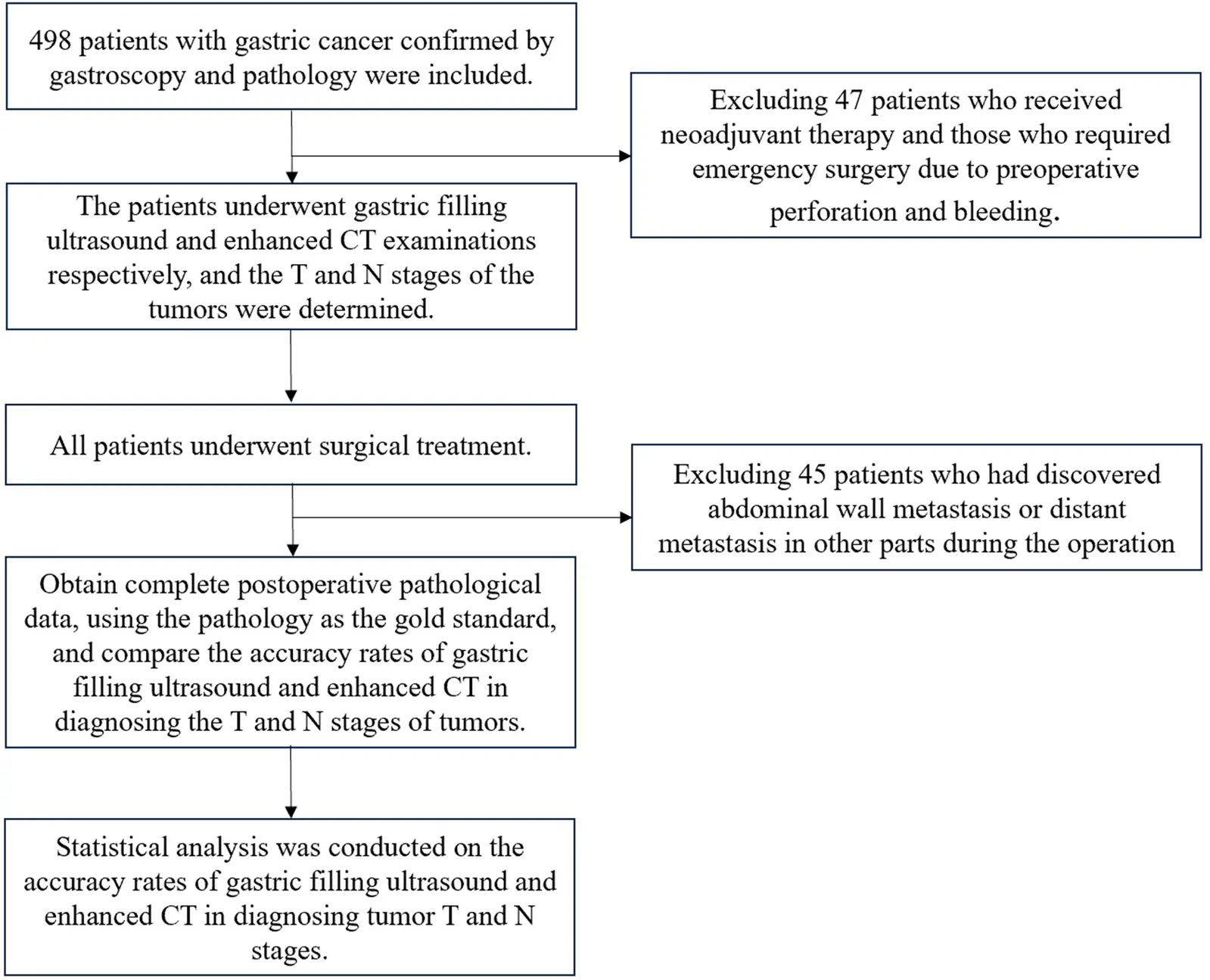
The flowchart of this study.

### Scanning methods

#### CT acquisition protocol

Gastric-enhanced CT was performed using a 64-row scanner (Brilliance 64, Philips Healthcare, the Netherlands) either one day before or after gastric filling ultrasonography. Patients fasted for at least 6 h and ingested 600–1,200 mL of water immediately prior to the examination. All scans were acquired in the supine position, extending from the diaphragmatic dome to the anterior superior iliac spine. The scanning parameters are set as follows: tube voltage: 120 kV, tube current: 300 mAs, slice thickness: 5 mm, slice interval: 5 mm. Non-contrast scanning was followed by intravenous administration of 90 mL of iohexol (Omnipaque, GE Healthcare) at a flow rate of 3 mL/s via the antecubital vein, immediately flushed with 30 mL of normal saline. Arterial-phase and venous-phase images were obtained at 30 s and 70 s after contrast injection, respectively.

#### Ultrasound imaging protocol

Gastric filling ultrasonography was scheduled on a separate day—either the day before or after CT—to avoid potential interference between the two examinations. Patients were instructed to fast for at least 8 h and refrain from liquid intake for 4 h prior to the examination. On the evening before the examination, patients were advised to consume easily digestible meals and avoid gas-producing or high-fat foods. The oral contrast agent (Yanbian Junyi Medical Technology Co., Ltd., Changchun, China) was freshly prepared by mixing approximately 500 mL of hot water (90–100 °C) with the contrast powder, with the total volume adjusted according to individual patient tolerance. The frequency setting of the ultrasound probe used in this study was 3.5–5 MHz. A standardized scanning protocol was followed, encompassing five standard anatomic views of the stomach in a predefined sequence: the lower esophagus and cardia, the fundus, the greater and lesser curvatures, the anterior and posterior walls, the gastric angle, the antrum, and the pylorus to the duodenal bulb. The transducer was moved slowly and continuously across all sections to ensure complete visualization without overlooking any region. No serious adverse events including vomiting or aspiration were observed. Mild transient abdominal distension was reported in a small subset of participants, but no subject discontinued the examination due to intolerance.

### Image interpretation

Ultrasound images were independently reviewed by two senior physicians, each with over 10 years of experience in abdominal ultrasonography, who were blinded to the pathological and CT findings. Each reviewer independently assigned T and N categories. Any discrepancies were resolved through consensus discussion. Similarly, gastric-enhanced CT images were independently assessed by two senior radiologists who were blinded to the ultrasound and pathological data, with T and N stages recorded for each modality.

### Staging criteria

We use the 8th edition of AJCC TNM for staging, as follows:

*T1 stage*: On gastric filling ultrasound, the stomach wall retains its normal five-layer appearance, with localized thickening of the mucosal layer but no abrupt interruption of the inner or outer layers. The interface between the lesion and the muscularis propria (submucosal layer) remains intact, without evidence of infiltration. On gastric-enhanced CT, the tumor is confined to the mucosa, and the gastric wall may not appear thickened; the normal mucosa may demonstrate contrast enhancement.

*T2 stage*: Ultrasound shows transmural hyperechoic change with localized thickening of the gastric wall. The outer margin of the thickened wall is smooth, with clear fat-plane separation from adjacent organs. On gastric-enhanced CT, tumor invasion extends into the muscularis propria.

*T3 stage*: Ultrasound reveals thickening of the gastric wall with an irregular, coarse serosal surface. Nodular or band-like echogenicities are observed in the perigastric fat space. On gastric-enhanced CT, the tumor invades beyond the muscularis into the subserosal layer.

*T4 stage*: Ultrasound examination reveals thickening of the stomach wall, disappearance of the fat layer between the stomach and adjacent organs, suggesting direct invasion or penetration of the serosal layer. Gastric-enhanced CT shows that the tumor has invaded adjacent structures or has penetrated the serosal layer.

*N stage*: Based on the number of metastatic lymph nodes, we classify the absence of regional lymph node metastasis as N0, 1–2 regional lymph node metastases as N1, 3–6 as N2, and ≥7 as N3.

### Statistical analysis

SPSS 25.0 statistical software was used for data analysis. Count data were expressed as frequency (*n*) and percentage (%). Since both gastric filling ultrasound and gastric-enhanced CT were performed on the same patient, comparisons of diagnostic accuracy (T-stage and N-stage) between the two paired modalities were analyzed using McNemar's test. A *P*-value < 0.05 was considered statistically significant. Receiver operating characteristic (ROC) curves were plotted, and the area under the curve (AUC) was calculated to assess diagnostic efficacy. The comparison of AUCs between the two modalities was further performed using the DeLong test. Sensitivity and specificity were calculated using standard formulas. Kappa statistics were calculated to measure the consistency of each method with the gold standard pathological results (0.8–1, almost perfect; 0.6–0.8, substantial; 0.4–0.6, moderate). Additionally, inter-observer agreement for ultrasound T-staging was assessed using Kappa coefficients. Factors influencing the accuracy of T-staging by gastric filling ultrasound were identified using univariate Logistic regression analysis.

## Results

### Clinical data

A total of 406 patients with complete postoperative pathological data and preoperative imaging (both gastric filling ultrasonography and gastric-enhanced CT) were enrolled in this study. The pathological T-stage distribution was as follows: T1, *n* = 119; T2, *n* = 53; T3, *n* = 140; and T4, *n* = 94. On gastric filling ultrasound, the majority of tumors across all T-stages appeared as hypoechoic lesions with ill-defined borders and disruption of the normal gastric wall layering.

As shown in Table 1, for gastric-enhanced CT -based T-staging, no statistically significant associations were observed between staging accuracy (or misclassification rate) and any of the clinicopathological variables examined. For CT-based N-staging, however, accuracy differed significantly according to gross type (*P* = 0.001), lymph node metastasis (*P* < 0.001), vascular infiltration (*P* < 0.001), perineural invasion (*P* < 0.001), and tumor size (*P* < 0.001).

**Table 1:** Comparison of clinicopathological characteristics between patients with accurate and inaccurate T- and N-staging by gastric-enhanced CT.

Variables		T-stage		Total (*n*)	***P***	N-stage		Total (*n*)	***P***
		[*n* (%)]				[*n* (%)]			
		No	Yes			No	Yes		
Age					0.290				0.613
	≤55	27 (18.24)	51 (19.77)	78		33 (20.37)	45 (18.44)	78	
	>55	121 (81.76)	207 (80.23)	328		129 (79.63)	199 (81.56)	328	
	Total (*n*)	148	258	406		162	244	406	
Sex					0.813				1.000
	Female	47 (31.76)	80 (31.01)	127		52 (32.10)	75 (30.74)	127	
	Male	101 (68.24)	178 (68.99)	279		110 (67.90)	169 (69.26)	279	
	Total (*n*)	148	258	406		162	244	406	
Degree of differentiation					0.625				0.061
	Poorly differentiated	56 (37.84)	102 (39.53)	158		67 (41.36)	91 (37.30)	158	
	Moderately differentiated	89 (60.14)	145 (56.20)	234		91 (56.17)	143 (58.61)	234	
	Well-differentiated	2 (1.35)	9 (3.49)	11		2 (1.23)	9 (3.69)	11	
	Undifferentiated	1 (0.68)	2 (0.78)	3		2 (1.23)	1 (0.41)	3	
	Total (*n*)	148	258	406		162	244	406	
Tumor size					0.195				<0.001
	≤2.5	33 (22.30)	58 (22.48)	91		21 (12.96)	70 (28.69)	91	
	>2.5	115 (77.70)	200 (77.52)	315		141 (87.04)	174 (71.31)	315	
	Total (*n*)	148	258	406		162	244	406	
Tumor location					0.909				0.301
	Cardia	13 (8.78)	24 (9.30)	38		18 (11.11)	20 (8.20)	38	
	Gastric fundus	5 (3.38)	10 (3.88)	15		4 (2.47)	11 (4.51)	15	
	Gastric antrum	74 (50.00)	142 (55.04)	215		83 (51.23)	132 (54.10)	215	
	Gastric angle	4 (2.70)	9 (3.49)	13		3 (1.85)	10 (4.10)	13	
	Gastric body	52 (35.14)	73 (28.29)	125		54 (33.33)	71 (29.10)	125	
	Total (*n*)	148	258	406		162	244	406	
Gross typing					0.159				0.001
	Early gastric cancer	46 (31.08)	82 (31.78)	128		32 (19.75)	96 (39.34)	128	
	Polyp type	1 (0.68)	7 (2.71)	8		3 (1.85)	5 (2.05)	8	
	Ulcer localized type	14 (9.46)	16 (6.20)	30		9 (5.56)	21 (8.61)	30	
	Ulcer infiltrating type	74 (50.00)	128 (49.61)	202		100 (61.73)	102 (41.80)	202	
	Diffuse infiltrating type	13 (8.78)	25 (9.69)	38		18 (11.11)	20 (8.20)	38	
	Total (*n*)	148	258	406		162	244	406	
Pathological types					0.769				0.125
	Adenocarcinoma	143 (96.62)	249 (96.51)	392		154 (95.06)	238 (97.54)	392	
	Neuroendocrine carcinoma	1 (0.68)	1 (0.39)	2		0 (0.00)	2 (0.82)	2	
	Mucinous adenocarcinoma	4 (2.70)	5 (1.94)	9		7 (4.32)	2 (0.82)	9	
	Adenosquamous carcinoma	0 (0.00)	1 (0.39)	1		0 (0.00)	1 (0.41)	1	
	Medullary carcinoma	0 (0.00)	2 (0.78)	2		1 (0.62)	1 (0.41)	2	
	Total	148	258	406		162	244	406	
Lymph node metastasis					0.367				<0.001
	No	67 (45.27)	101 (39.15)	168		44 (27.16)	124 (50.82)	168	
	Yes	81 (54.73)	157 (60.85)	238		118 (72.84)	120 (49.18)	238	
	Total (*n*)	148	258	406		162	244	406	
Abdominal wall thickness					0.597				0.577
	≤2.5	111 (75.00)	221 (85.66)	338		124 (76.54)	214 (87.70)	338	
	>2.5	31 (20.95)	37 (14.34)	68		38 (23.46)	30 (12.30)	68	
	Total (*n*)	148	258	406		162	244	406	
Vascular infiltration					0.324				<0.001
	No	65 (43.92)	93 (36.05)	158		39 (24.07)	119 (48.77)	158	
	Yes	83 (56.08)	165 (63.95)	248		123 (75.93)	125 (51.23)	248	
	Total (*n*)	148	258	406		162	244	406	
Perineural invasion					1.000				<0.001
	No	68 (45.95)	121 (46.90)	189		54 (33.33)	135 (55.33)	189	
	Yes	80 (54.05)	137 (53.10)	217		108 (66.67)	109 (44.67)	217	
	Total (*n*)	148	258	406		162	244	406	

As shown in Table 2, for ultrasound-based T-staging, abdominal wall thickness was the only variable significantly associated with staging accuracy (*P* = 0.001). For ultrasound-based N-staging, accuracy varied significantly by sex (*P* = 0.002), lymph node metastasis (*P* = 0.007), and vascular infiltration (*P* = 0.049).

**Table 2:** Comparison of clinicopathological characteristics between patients with accurate and inaccurate T- and N-staging by gastric-filling ultrasonography.

Variables		T-stage		Total (*n*)	*P*	N-Stage		Total (*n*)	*P*
		[*n* (%)]				[*n* (%)]			
		No	Yes			No	Yes		
Age					0.348				0.505
	≤55	18 (16.22)	60 (20.34)	78		12 (16.44)	66 (19.82)	78	
	>55	93 (83.78)	235 (79.66)	328		61 (83.56)	267 (80.18)	328	
	Total (*n*)	111	295	406		73	333	406	
Sex					0.869				0.002
	Female	34 (30.63)	93 (31.53)	127		34 (46.58)	93 (27.93)	127	
	Male	77 (69.37)	202 (68.47)	279		39 (53.42)	240 (72.07)	279	
	Total (*n*)	111	295	406		73	333	406	
Degree of differentiation					0.694				0.166
	Poorly differentiated	38 (34.23)	120 (40.68)	158		26 (35.62)	132 (39.64)	158	
	Moderately differentiated	69 (62.16)	165 (55.93)	234		43 (58.90)	191 (57.36)	234	
	Well-differentiated	3 (2.70)	8 (2.71)	11		2 (2.74)	9 (2.70)	11	
	Undifferentiated	1 (0.90)	2 (0.68)	3		2 (2.74)	1 (0.30)	3	
	Total (*n*)	111	295	406		73	333	406	
Tumor size					0.975				0.845
	≤2.5	25 (22.52)	66 (22.37)	91		17 (23.29)	74 (22.22)	91	
	>2.5	86 (77.48)	229 (77.63)	315		56 (76.71)	259 (77.78)	315	
	Total (*n*)	111	295	406		73	333	406	
Tumor location					0.793				0.562
	Cardia	11 (9.91)	27 (9.15)	38		7 (9.59)	31 (9.31)	38	
	Gastric fundus	4 (3.60)	11 (3.73)	15		5 (6.85)	10 (3.00)	15	
	Gastric antrum	61 (54.95)	154 (52.20)	215		38 (52.05)	177 (53.15)	215	
	Gastric angle	5 (4.50)	8 (2.71)	13		3 (4.11)	10 (3.00)	13	
	Gastric body	30 (27.03)	95 (32.20)	125		20 (27.40)	105 (31.53)	125	
	Total (*n*)	111	295	406		73	333	406	
Gross typing					0.144				0.051
	Early gastric cancer	25 (22.52)	103 (34.92)	128		18 (24.66)	110 (33.03)	128	
	Polyp type	3 (2.70)	5 (1.69)	8		4 (5.48)	4 (1.20)	8	
	Ulcer localized type	7 (6.31)	23 (7.80)	30		8 (10.96)	22 (6.61)	30	
	Ulcer infiltrating type	64 (57.66)	138 (46.78)	202		34 (46.58)	168 (50.45)	202	
	Diffuse infiltrating type	12 (10.81)	26 (8.81)	38		9 (12.33)	29 (8.71)	38	
	Total (*n*)	111	295	406		73	333	406	
Pathological types					0.161				0.237
	Adenocarcinoma	106 (95.50)	286 (96.95)	392		70 (95.89)	322 (96.70)	392	
	Neuroendocrine carcinoma	0 (0.00)	2 (0.68)	2		0 (0.00)	2 (0.60)	2	
	Mucinous adenocarcinoma	3 (2.70)	6 (2.03)	9		2 (2.74)	7 (2.10)	9	
	Adenosquamous carcinoma	0 (0.00)	1 (0.34)	1		1 (1.37)	0 (0.00)	1	
	Medullary carcinoma	2 (1.80)	0 (0.00)	2		0 (0.00)	2 (0.60)	2	
	Total	111	295	406		73	333	406	
Lymph node metastasis					0.178				0.007
	No	40 (36.04)	128 (43.39)	168		20 (27.40)	148 (44.44)	168	
	Yes	71 (63.96)	167 (56.61)	238		53 (72.60)	185 (55.56)	238	
	Total (*n*)	111	295	406		73	333	406	
Abdominal wall thickness					0.001				0.951
	≤2.5	81 (72.97)	257 (87.12)	338		61 (83.56)	277 (83.18)	338	
	>2.5	30 (27.03)	38 (12.88)	68		12 (16.44)	56 (16.82)	68	
	Total (*n*)	111	295	406		73	333	406	
Vascular infiltration					0.036				0.049
	No	34 (30.63)	124 (42.03)	124 (41.81) 158		21 (28.77)	137 (41.14)	158	
	Yes	77 (69.37)	171 (57.97)	248		52 (71.23)	196 (58.86)	248	
	Total (*n*)	111	295	406		73	333	406	
Perineural Invasion					0.054				0.121
	No	43 (38.74)	146 (49.49)	189		28 (38.36)	161 (48.35)	189	
	Yes	68 (61.26)	149 (50.51)	217		45 (61.64)	172 (51.65)	217	
	Total (*n*)	111	295	406		73	333	406	

### Comparison of the accuracy of gastric-enhanced CT and gastric filling ultrasound for T and N staging assessment

#### T-stage accuracy

Compared with postoperative pathological findings, the overall accuracy of gastric filling ultrasonography for preoperative T-staging was 72.7% (295/406), vs. 63.5% (258/406) for gastric-enhanced CT. Stage-specific accuracy for ultrasound vs. CT was as follows: T1, 81.5% (97/119) vs. 56.3% (67/119); T2, 81.1% (43/53) vs. 67.9% (36/53); T3, 72.1% (101/140) vs. 65.0% (91/140); and T4, 57.4% (54/94) vs. 68.1% (64/94) (Tables 3 and 4). Ultrasound demonstrated significantly higher accuracy than CT for T1 (*P* = 0.009) and T2 (*P* < 0.001) staging. For T3 lesions, ultrasound also showed a modest advantage, though the difference did not reach statistical significance (*P* = 0.409). For T4 staging, CT outperformed ultrasound, but again the difference was not statistically significant (*P* = 0.319) (Supplementary Table 1).

**Table 3:** Accuracy of gastric-enhanced CT for assessment of individual T stages.

Pathologic T-staging	*n*	T-stage (*n*)				
		T0	T1	T2	T3	T4
T1	119	14	67	18	18	2
T2	53	—	1	36	10	6
T3	140	—	2	10	91	37
T4	94	—	—	1	29	64
Total (*n*)	406	14	70	65	148	109

**Table 4:** Accuracy of gastric filling ultrasound for assessment of individual T stages.

Pathologic T-staging	*n*	T-Stage (n)				
		T0	T1	T2	T3	T4
T1	119	—	97	17	5	—
T2	53	—	2	43	8	—
T3	140	—	1	6	101	32
T4	94	—	—	3	37	54
Total (*n*)	406	—	100	69	151	86

#### N-stage accuracy

For N-staging, the overall accuracy of gastric filling ultrasound was 82.0% (333/406), substantially higher than that of gastric-enhanced CT (60.1%, 244/406). Stage-specific accuracy for ultrasound vs. CT was: N0, 90.4% (151/167) vs. 86.8% (145/167); N1, 83.6% (51/61) vs. 44.3% (27/61); N2, 75.8% (47/62) vs. 48.4% (30/62); and N3, 76.7% (89/116) vs. 36.2% (42/116) (Tables 5 and 6). The two modalities performed comparably in N0 staging, with no significant difference between them (*P* = 0.267). For N1 through N3, however, ultrasound was significantly more accurate than CT (*P* = 0.004, *P* = 0.042, and *P* < 0.001, respectively) (Supplementary Table 2).

**Table 5:** Accuracy of gastric filling ultrasound for assessment of individual N stages.

Pathologic N-staging	*n*	N-stage (*n*)			
		N0	N1	N2	N3
N0	167	151	14	2	—
N1	61	9	51	1	—
N2	62	5	6	47	4
N3	116	3	11	13	89
Total (*n*)	406	168	82	63	93

**Table 6:** Accuracy of gastric-enhanced CT for assessment of individual N stages.

Pathologic N-staging	*n*	N-stage (*n*)			
		N0	N1	N2	N3
N0	167	145	15	6	1
N1	61	27	27	5	2
N2	62	11	20	30	1
N3	116	22	26	26	42
Total (*n*)	406	205	88	67	46

### Diagnostic efficacy of gastric filling ultrasound and gastric-enhanced CT for each T-stage gastric cancer

ROC curves were constructed to compare the diagnostic performance of gastric filling ultrasonography and enhanced CT for each T category, with corresponding AUC values calculated for both modalities (Figure 2). For T1 staging, ultrasound achieved a sensitivity of 81.5% and specificity of 99.0%, compared with 56.3% and 99.0% for CT. The AUC for ultrasound was 0.902, significantly higher than that for CT (0.776; *Z* = 2.45, *P* = 0.014). For T2 staging, ultrasound yielded a sensitivity of 81.1% and specificity of 92.6% (AUC = 0.869), whereas CT produced 67.9% and 91.8%, respectively (AUC = 0.799; *Z* = 2.18, *P* = 0.029). For T3 staging, ultrasound demonstrated a sensitivity of 72.1% and specificity of 81.2% (AUC = 0.767), compared with 65.0% and 78.6% for CT (AUC = 0.718). For T4 staging, ultrasound showed a sensitivity of 57.4% and specificity of 89.7% (AUC = 0.736), while CT exhibited 68.1% and 85.6% (AUC = 0.768); the difference between the two AUCs was not statistically significant (*Z* = 0.87, *P* = 0.384). The DeLong test also indicated no significant difference between the two modalities for T3 staging (*P* > 0.05).

**Figure 2:**
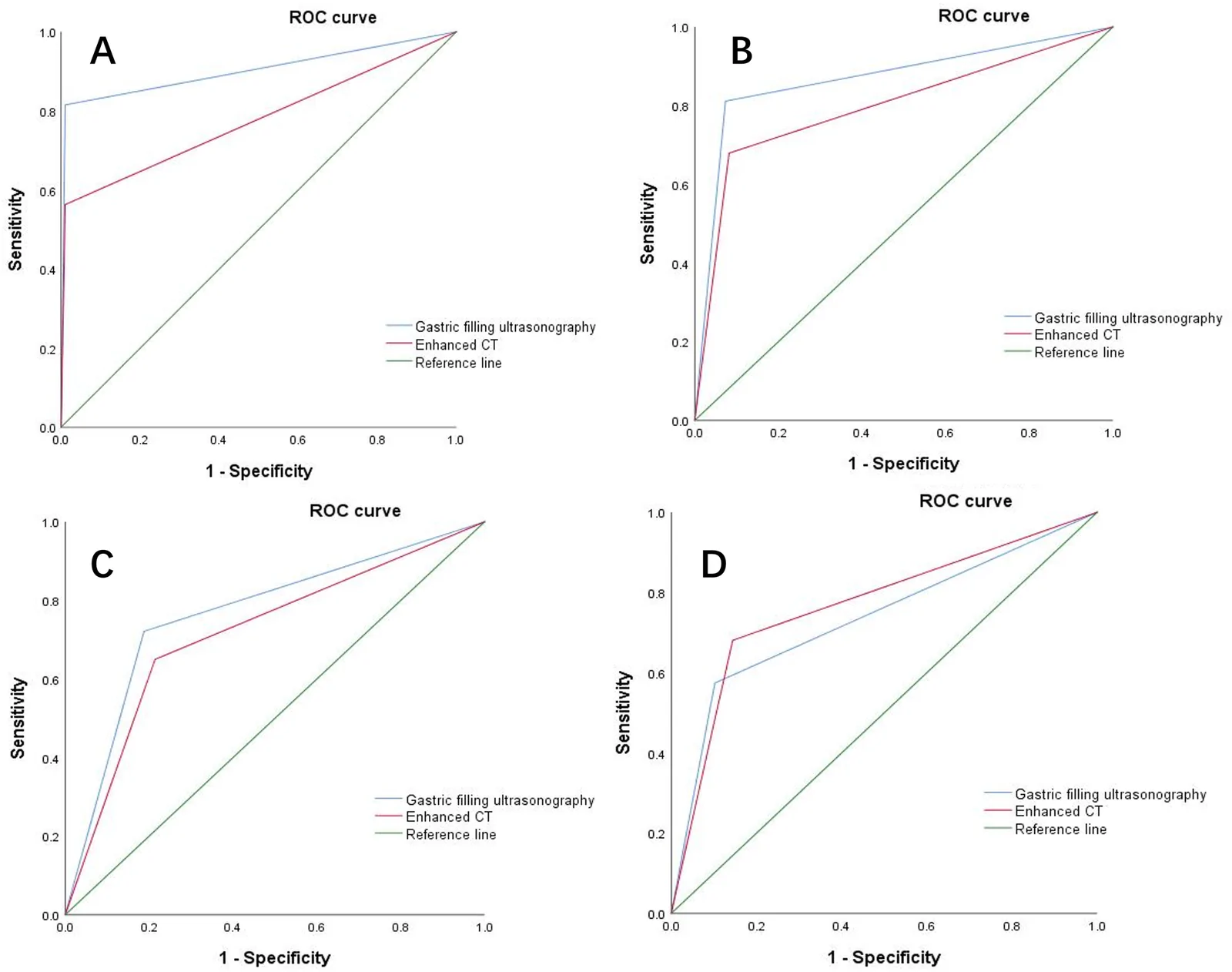
**(A)** ROC curves of gastric filling ultrasound and gastric-enhanced CT for T1 stage. **(B)** ROC curves of gastric filling ultrasound and gastric-enhanced CT for T2 stage. **(C)** ROC curves of gastric filling ultrasound and gastric-enhanced CT for T3 stage. **(D)** ROC curves of gastric filling ultrasound and gastric-enhanced CT for T4 stage. ROC, receiver operating characteristic curve.

Agreement with pathological T-staging, as measured by unweighted Kappa, was substantial for ultrasound (*κ* = 0.625; 95% CI: 0.566–0.684) and moderate for CT (*κ* = 0.508; 95% CI: 0.445–0.571). Using linear-weighted Kappa accounting for ordinal staging misclassification, ultrasound achieved *κ* = 0.681 (95%CI: 0.624–0.738), while CT yielded *κ* = 0.552 (95%CI: 0.490–0.614). For T-staging, exact agreement rate was 72.7% for ultrasound vs. 63.5% for CT; under-staging rate was 14.0% vs. 19.2%, and over-staging rate was 13.3% vs. 17.2%.

### Diagnostic efficacy of gastric filling ultrasound and gastric-enhanced CT for each N-stage gastric cancer

ROC curves were constructed to evaluate the diagnostic performance of gastric filling ultrasonography and enhanced CT for each N category, and the corresponding AUC values were calculated (Figure 3). For N0 staging, ultrasound achieved a sensitivity of 90.4% and specificity of 92.9% (AUC = 0.902), compared with 78.4% and 74.9% for CT (AUC = 0.767). For N1 staging, ultrasound yielded a sensitivity of 83.6% and specificity of 90.1% (AUC = 0.869), whereas CT produced 44.3% and 82.3%, respectively (AUC = 0.633). For N2 staging, ultrasound demonstrated a sensitivity of 75.8% and specificity of 95.1% (AUC = 0.854), compared with 48.4% and 89.2% for CT (AUC = 0.688). For N3 staging, ultrasound showed a sensitivity of 76.7% and specificity of 98.3% (AUC = 0.875), while CT exhibited 36.2% and 98.6% (AUC = 0.674).

**Figure 3:**
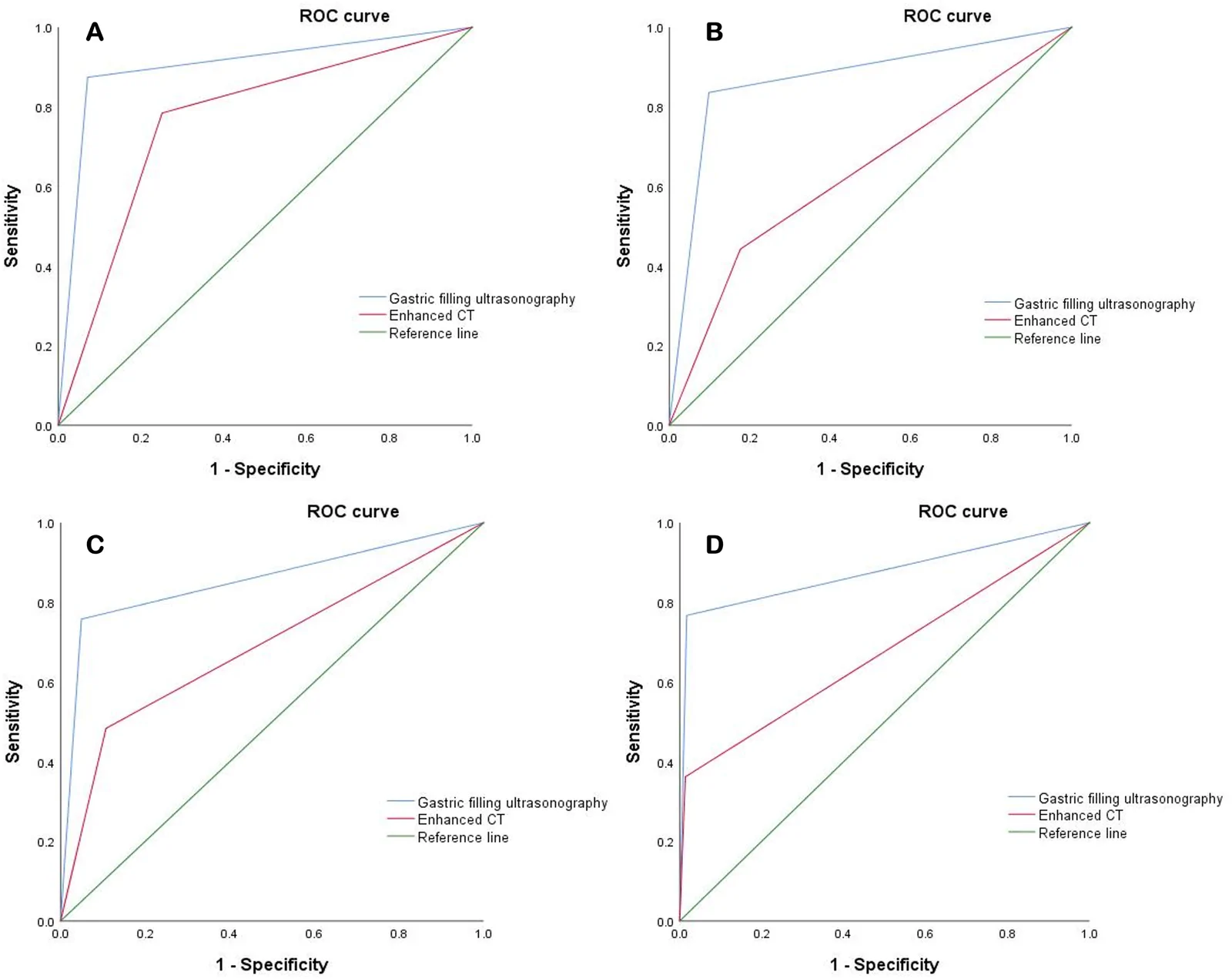
**(A)** ROC curves of gastric filling ultrasound and gastric-enhanced CT for N0 stage. **(B)** ROC curves of gastric filling ultrasound and gastric-enhanced CT for N1 stage. **(C)** ROC curves of gastric filling ultrasound and gastric-enhanced CT for N2 stage. **(D)** ROC curves of gastric filling ultrasound and gastric-enhanced CT for N3 stage. ROC, receiver operating characteristic curve.

Agreement with pathological N-staging, as measured by unweighted Kappa, was substantial for ultrasound (*κ* = 0.748; 95% CI: 0.697–0.799) and moderate for CT (*κ* = 0.395; 95% CI: 0.334–0.456). The linear-weighted Kappa for N-staging was *κ* = 0.773 (95%CI: 0.723–0.823) for ultrasound and *κ* = 0.441 (95%CI: 0.378–0.504) for CT. For N-staging, exact agreement rate was 82.0% for ultrasound vs. 60.1% for CT; under-staging rate was 9.9% vs. 22.7%, and over-staging rate was 8.1% vs. 17.2%.

### Analysis of the accuracy of preoperative T and N staging of gastric filling ultrasound vs. gastric-enhanced CT

As shown in Table 7, in the subgroup of early gastric cancer (The cancerous tissue is confined to the mucosal layer and submucosal layer of the stomach, regardless of its size and whether there is lymph node metastasis), ultrasound demonstrated significantly higher T-staging accuracy than CT for tumors located in the gastric body with a diameter >2.5 cm.

**Table 7:** Comparison of the accuracy of gastric filling ultrasound and gastric-enhanced CT for T-staging assessment.

Variables	Total (*n*)	Gastric filling ultrasonography	Gastric-enhanced CT	*P*
		*n* (%)	*n* (%)	
Gross typing				
Early gastric cancer	128	103 (80.5)	82 (64.1)	0.005
Polyp type	8	5 (62.5)	7 (87.5)	0.564
Ulcer localized type	30	23 (76.7)	16 (53.3)	0.104
Ulcer infiltrating type	202	138 (68.3)	128 (63.4)	0.345
Diffuse infiltrating type	38	26 (68.4)	25 (65.8)	1.000
Tumor location				
Cardia	38	27 (71.1)	24 (63.2)	0.625
Gastric fundus	15	11 (73.3)	10 (66.7)	1.000
Gastric antrum	215	154 (71.6)	142 (66.0)	0.252
Gastric angle	13	8 (61.5)	9 (69.2)	1.000
Gastric body	125	95 (76.0)	73 (58.4)	0.005
Tumor size				
≤2.5 cm	91	66 (72.5)	58 (63.7)	0.266
>2.5 cm	315	229 (72.7)	200 (63.5)	0.017

Early gastric cancer was defined by pathological T stage. *P* values were calculated using McNemar's test for paired data.

As shown in Table 8, for early gastric cancer and ulcer-infiltrating type gastric cancer, ultrasound achieved significantly higher N-staging accuracy than CT in the following subgroups: tumors located at the cardia, antrum, or gastric body, and tumors with a diameter >2.5 cm.

**Table 8:** Comparison of the accuracy of gastric filling ultrasound and gastric-enhanced CT for N-staging assessment.

Variables	Total (*n*)	Gastric filling ultrasonography	Gastric-enhanced CT	*P*
		*n* (%)	*n* (%)	
Gross typing				
Early gastric cancer	128	110 (85.9)	96 (75.0)	0.040
Polyp type	8	4 (50.0)	5 (62.5)	1.000
Ulcer localized type	30	22 (73.3)	21 (70.0)	1.000
Ulcer infiltrating type	202	168 (83.2)102 (50.5)	102 (50.5)	<0.001
Diffuse infiltrating type	38	29 (76.3)20 (52.6)	20 (52.6)	0.055
Tumor location				
Cardia	38	31 (81.6)	20 (52.6)	0.015
Gastric fundus	15	10 (66.7)	11 (73.3)	1.000
Gastric antrum	215	177 (82.3)	132 (61.4)	<0.001
Gastric angle	13	10 (76.9)	10 (76.9)	1.000
Gastric body	125	105 (84.0)	71 (56.8)	<0.001
Tumor size				
≤2.5 cm	91	74 (81.3)	70 (76.9)	0.584
>>2.5 cm	315	259 (82.2)	174 (55.2)	<0.001

Early gastric cancer was defined by pathological T stage. *P* values were calculated using McNemar's test for paired data.

### Exploratory analysis of factors influencing T-staging accuracy of gastric-filling ultrasonography

To explore factors associated with the accuracy of gastric-filling ultrasonography for T-staging, we performed exploratory univariate and multivariate logistic regression analyses. Variables with *P*  < 0.05 in univariate analysis were entered into multivariate regression for confounding adjustment. Detailed regression outputs are presented in Supplementary Tables 5, 6. Univariate regression results were shown in Supplementary Table 5. Three variables met the screening criteria of *P* < 0.05: vascular infiltration (*P* = 0.036), perineural invasion (*P* = 0.044), and gross typing (*P* = 0.037). Other variables showed no significant association with the accuracy of ultrasound T-staging (*P* > 0.05). In multivariate analysis, vascular infiltration was the only independent factor associated with ultrasound T-staging accuracy (OR = 0.612, 95%CI: 0.374–0.999, *P* = 0.049). Perineural invasion and gross typing showed no statistical significance after adjustment. we constructed a stratified subgroup model to screen the preferred patient population suitable for preoperative T-staging by gastric-filling ultrasonography. The inclusion criteria for the subgroup were: (1) gross typing: early gastric cancer or polyp type; (2) tumor size ≤2.5 cm; (3) absence of vascular infiltration; (4) absence of perineural invasion; (5) abdominal wall thickness >2.5 cm. A total of 64 patients were enrolled in this subgroup. The dataset was randomly split into a training set (*n* = 45, 70%) and a test set (*n* = 19, 30%) using stratified sampling. In the selected preferred subgroup (*n* = 64), the prediction model reached a test-set accuracy of 96.88% and AUC of 0.912, test set sensitivity is 94.12% and specificity is 96.04%.

Representative images of gastric tumors at each T stage, as visualized by both gastric filling ultrasonography and gastric-enhanced CT, are presented in Figures 4–8.

**Figure 4:**
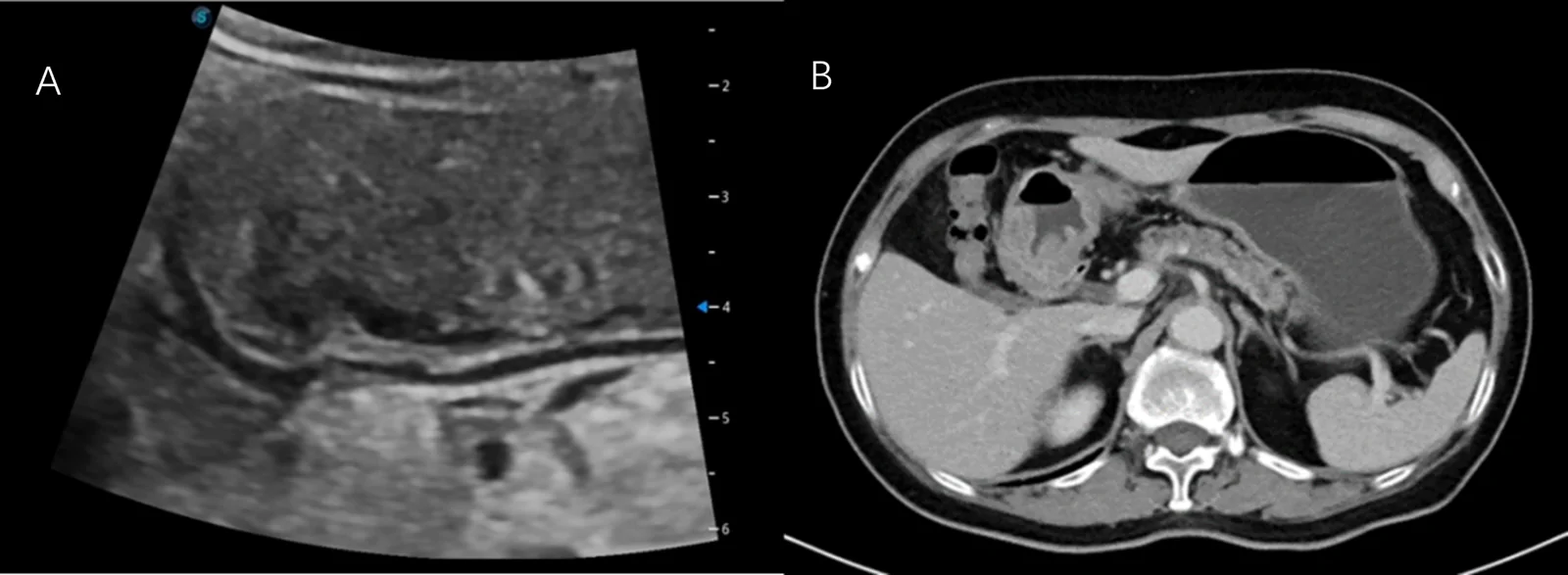
**(A)** stage T1 tumors resemble the normal gastric wall on gastric filling ultrasound images, with localized thickening of the mucosal layer of the gastric wall, but without abrupt interruption of the middle and outer layers, and with a clear demonstration of the area between the base of the lesion and the muscular propria (equivalent to the submucosal layer), without infiltration. **(B)** gastric-enhanced CT images show that the tumor is confined to the mucosal layer, the gastric wall may not be thickened, and the normal mucosa may be enhanced.

**Figure 5:**
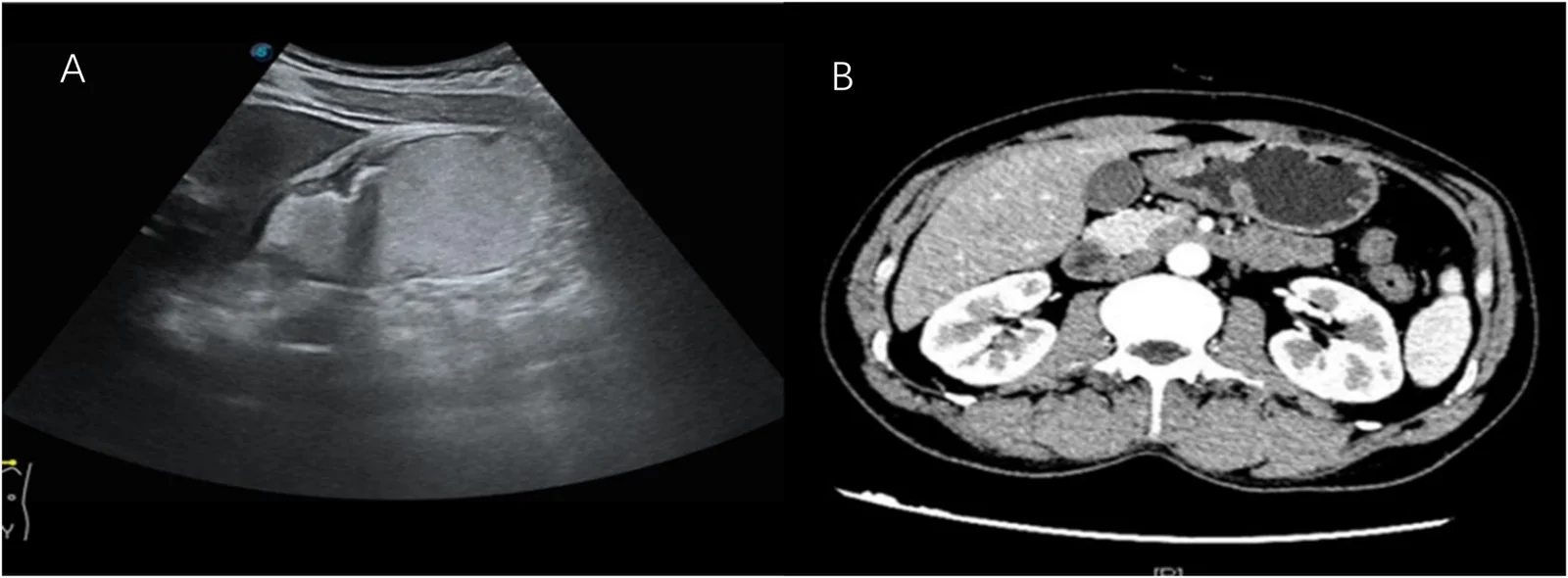
**(A)** T2 stage tumors in gastric filling ultrasound can be observed in the gastric wall. See transmural hyperechoic change with localized thickening and focal thickening of the outer edge of the gastric wall, which is glossy, and the fat layer between the adjacent organs is clear. **(B)** Gastric-enhanced CT shows invasion of the tumor lesion into the muscularis propria.

**Figure 6:**
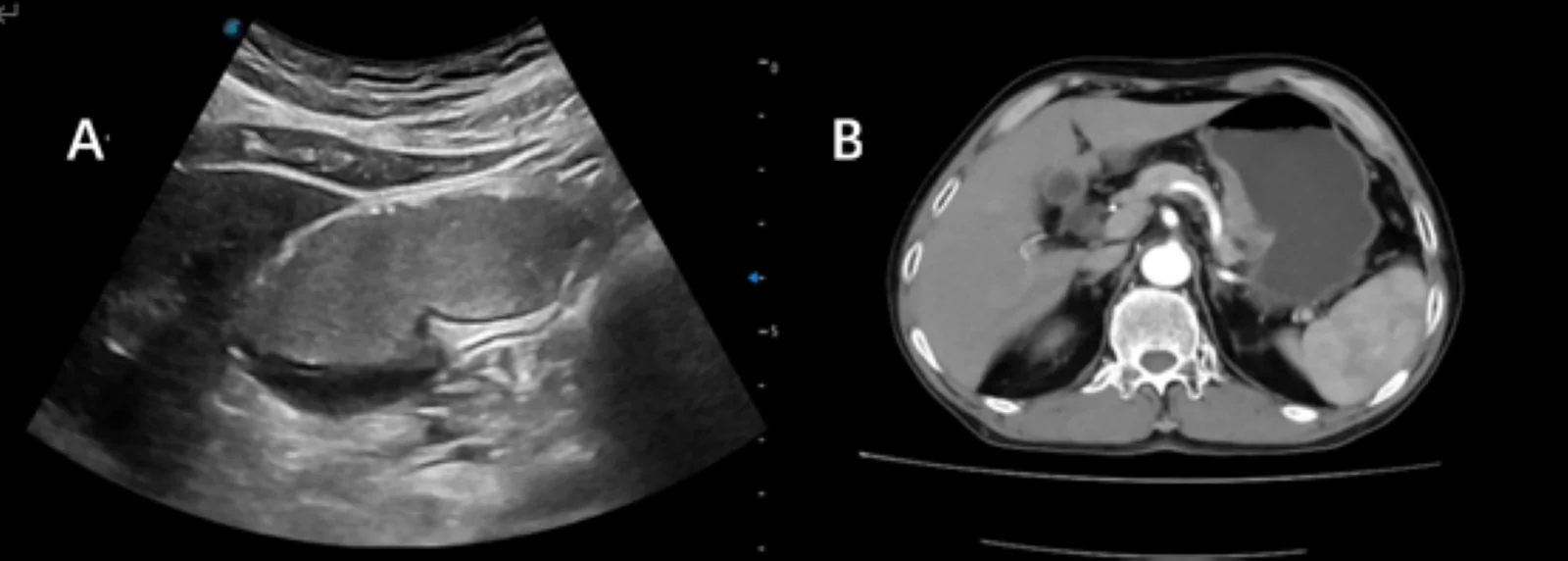
**(A)** T3 stage tumors are seen on gastric filling ultrasound as thickening of the gastric wall with a gross, the surface of the serous membrane shows coarse and irregular changes. **(B)** Gastric-enhanced CT shows that the tumor lesion is invading the serous membrane.

**Figure 7:**
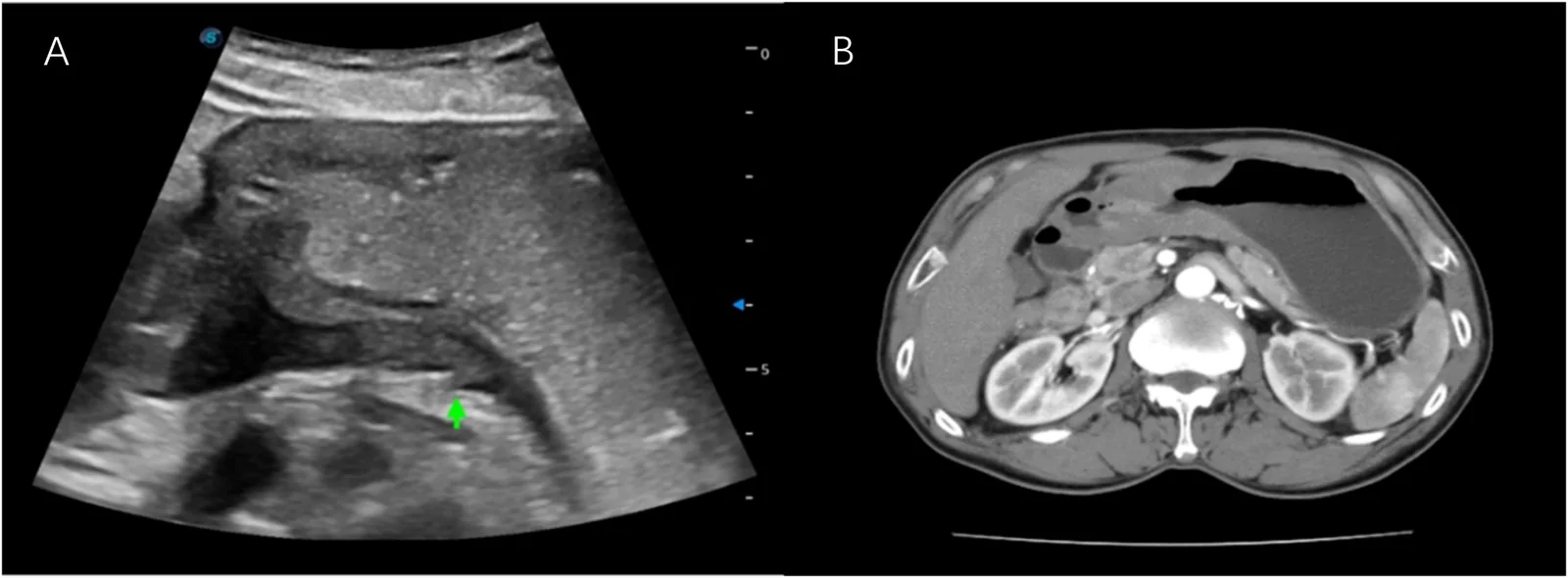
**(A)** T4 stage gastric cancer in gastric filling ultrasound may observe thickening of the gastric wall, disappearance of the fatty interstitial space between adjacent organs, and tumor invasion of neighboring organs. **(B)** Gastric-enhanced CT shows tumor invasion into other organs.

**Figure 8:**
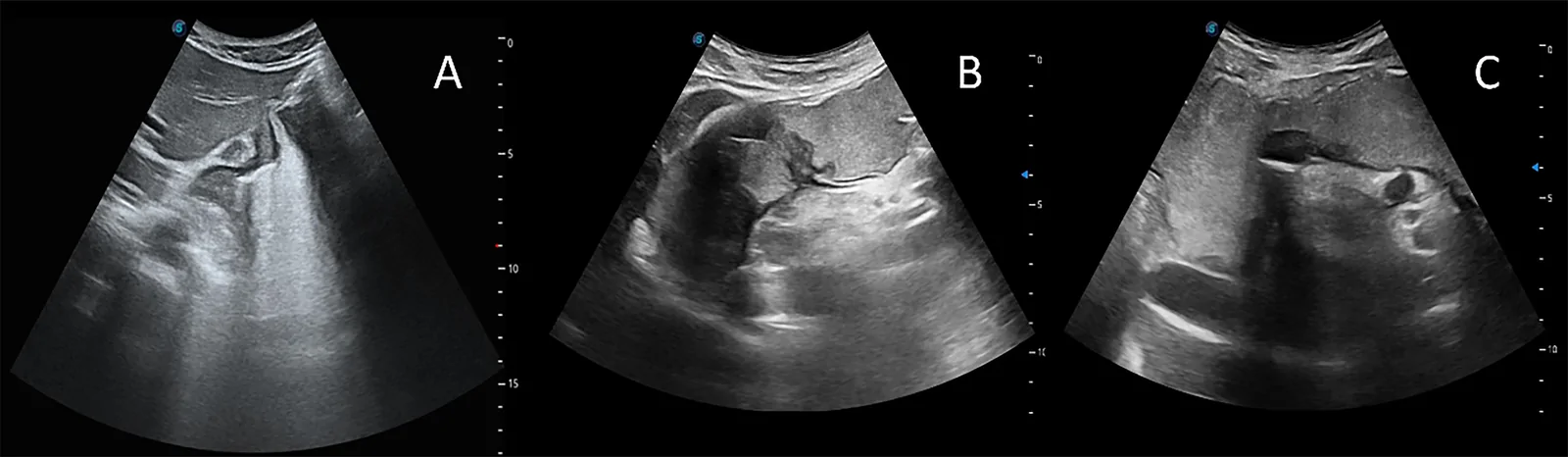
**(A)** Ultrasound image of lymph nodes at the cardia. **(B)** Ultrasound image of lymph nodes at the gastric antrum. **(C)** Ultrasound image of lymph nodes at the lesser curvature of the stomach.

## Discussion

Accurate preoperative TNM staging is critical in gastric cancer management, as it directly informs the selection of appropriate treatment strategies. Although postoperative histopathology remains the definitive staging reference, reliable imaging before surgery can substantially improve clinical decision-making. In this study, we used an oral echogenic contrast agent to achieve homogeneous gastric luminal distension, which enabled clear visualization of the gastric wall architecture and its five-layer structure. By assessing the integrity of these layers, tumor size, morphology, marginal characteristics, relationship with adjacent tissues, and regional lymph nodes, we were able to determine tumor extent and assign preoperative stage. Gastric filling ultrasonography offers several practical advantages: it is quick, non-invasive, inexpensive, radiation-free, and permits real-time dynamic observation of gastric peristalsis. These features have facilitated its progressive acceptance in clinical practice (12). Previous reports have shown that the detection rate of gastric filling ultrasound for gastric cancer is comparable to that of endoscopy (100% vs. 99.2%), suggesting its potential as a complementary screening tool, particularly for patients who cannot tolerate or decline invasive endoscopic examination.

In this prospective cohort, the overall diagnostic accuracy of gastric filling ultrasound for T-staging was superior to that of gastric-enhanced CT (72.7% vs. 63.5%). This advantage was most pronounced in early-stage tumors: ultrasound correctly identified 97 of 119 T1 lesions, whereas CT identified only 67. This disparity likely reflects the higher mural resolution of ultrasound following oral contrast administration, which allows detection of subtle localized thickening that may be imperceptible on CT, particularly when edema obscures the 50% wall thickness criterion often used in CT interpretation (13). Inadequate gastric distension further impairs CT assessment of mural layers, while the oral contrast agent used in ultrasound effectively eliminates intraluminal gas and contents, enabling detailed visualization of the all the layers of the stomach wall, delineation of their interface. Conversely, CT offers superior spatial resolution and is less susceptible to tumor location, providing robust assessment of deep infiltration and involvement of structures such as the peritoneum and diaphragm through evaluation of tumor vascularity and adjacent fat stranding (14).

Based on our stratified analysis, we propose a practical imaging workflow for gastrointestinal surgeons. For tumors located in the gastric antrum or body with a diameter <2.5 cm, gastric filling ultrasound should be strongly considered as the primary staging tool, given its high negative predictive value for deep invasion. In contrast, for tumors at the cardia or fundus, gastric-enhanced CT remains indispensable for evaluating potential diaphragmatic and peritoneal involvement. These two modalities should be viewed not as competitors, but as complementary components of a comprehensive staging approach. The ultrasound has a high accuracy rate (over 81%) in the T1–2 stage, which can reliably identify patients suitable for earlier T staging. Meanwhile, CT can effectively rule out potential T4 stage diseases.

While most previous studies comparing these two modalities for preoperative staging of advanced gastric cancer have been retrospective in design, the prospective nature of our study, which included patients across all stage categories, provides a more comprehensive and balanced evidence base. For T1 and T2 stages, ultrasound demonstrated significantly higher accuracy than CT (*P* = 0.009 and *P* < 0.001, respectively). For T3 lesions, ultrasound maintained a modest advantage, though this did not reach statistical significance in all subgroups. For T4 lesions, CT tended to outperform ultrasound, but the difference was not statistically significant (*P* > 0.05). It should be noted that the number of T2 cases in our series was relatively small, and further studies with larger samples are needed to substantiate the reliability of ultrasound in this specific subgroup. Nonetheless, the observed trend of ultrasound superiority in T1–T3 staging warrants consideration. The underlying mechanism may relate to the inflammatory response elicited by tumor invasion through the serosal and perigastric fat layers, which typically appears as hyperechogenicity on ultrasound; as penetration deepens, this echogenic signal diminishes and serosal continuity disruption becomes apparent (14).

In contrast, CT interpretation is frequently compromised by respiratory motion and subtle gastrointestinal peristalsis, both of which degrade image quality and blur mural detail. Additionally, peritumoral inflammation may manifest as hyperdense stranding on gastric-enhanced CT, mimicking serosal involvement and leading to overstaging (15, 16). On the other hand, ultrasound understaging may occur when lesions are small, when the hyperechoic serosal surface is difficult to differentiate from surrounding tissue, or when microscopic infiltration is present. Fibrosis at the base of tumor-associated ulcers can also obscure wall-layer architecture, and in some cases, the serosal layer may become indistinct or absent, contributing to staging inaccuracies. Technical factors, such as excessive abdominal fat, poor acoustic windows, or gas accumulation in the gastric fundus, further complicate ultrasound evaluation. These factors must be carefully weighed when interpreting ultrasound findings.

Regarding N-staging, ultrasound accuracy was significantly associated with sex (*P* = 0.003), gross tumor morphology (*P* = 0.033), and lymph node metastasis status (*P* = 0.011). For CT, staging accuracy correlated with gross tumor type (*P* = 0.001), lymph node metastasis (*P* < 0.001), vascular invasion (*P* < 0.001), perineural invasion (*P* < 0.001), and tumor size (*P* < 0.001). Tumor size is not merely a reflection of disease progression but is closely intertwined with lymph node status. Saito et al. noted that CT has limited sensitivity for detecting metastatic foci <5 mm, contributing to substantial rates of underdiagnosis and misclassification (17). CT may also erroneously categorize inflammatory lymphadenopathy as metastatic involvement, leading to discrepancies between imaging and pathological findings. For ultrasound, overstaging frequently arises from difficulty distinguishing inflammatory from metastatic nodes, especially when reactive enlargement is present. Micrometastases and small nodes located in the gastric fundus are particularly challenging to visualize due to gas interference and acoustic shadowing. Adherent nodal clusters with poorly defined borders further reduce diagnostic confidence. These observations underscore the inherent limitations of both modalities and highlight the need for adjunctive strategies to improve nodal evaluation.

In this cohort, the detection rate of early gastric cancer by gastric filling ultrasound reached 81.5%. Emerging evidence supports the feasibility of ultrasound-based screening in high-risk populations (9, 18, 19). Although endoscopy remains the gold standard for gastric cancer screening, its invasive nature limits patient acceptance, particularly among individuals with comorbidities or those reluctant to undergo the procedure. Compared with CT, MRI, or PET/CT, gastric filling ultrasound is radiation-free, cost-effective, and requires minimal preparation. However, it cannot provide histopathological confirmation, which constrains its role as a standalone diagnostic tool. Thus, for preoperative staging, these two examinations should be used in a complementary manner to enhance diagnostic accuracy, support informed clinical decisions, and guide individualized treatment planning.

In the present study, we constructed a preoperative prediction model which guaranteed the clinical applicability before surgery. Our multivariate analysis demonstrated that vascular infiltration was an independent risk factor for mis-T-staging by gastric-filling ultrasonography. Tumors with vascular infiltration often accompanied by submucosal micro-invasion and peritumoral inflammatory reaction, which may disrupt the layered structure of gastric wall and interfere with sonographic judgment of tumor invasion depth.

We performed subgroup stratification to identify the optimal candidate population for gastric-filling ultrasonography. Among patients met our subgroup inclusion criteria, the prediction model reached a test-set accuracy of 96.88%. This indicated that for patients with early or polyp-type tumor, small tumor size, absence of vascular and perineural infiltration and abdominal wall thickness >2.5 cm, gastric-filling ultrasonography could serve as a reliable primary preoperative T-staging tool. For patients outside this preferred subgroup, higher misdiagnosis risk should be alerted; complementary gastric-enhanced CT or endoscopic ultrasonography should be considered to optimize preoperative staging and clinical decision-making.

Several limitations of this study should be acknowledged. First, the sample was derived from a single tertiary center in Northeast China, which may introduce regional and demographic biases. Second, the distribution of patients across pathological stages was not entirely balanced, with relatively few T2 cases, potentially affecting the robustness of stage-specific comparisons. Third, image quality and diagnostic performance are operator- and equipment-dependent, and the influence of patient positioning, body habitus, and transducer selection warrants further investigation. Then, we did not collect independent interpretations from multiple readers during data acquisition; instead, we recorded consensus readings. As a result, we were unable to calculate inter-reader agreement, which limits the evaluation of reproducibility. Future diagnostic accuracy studies should prospectively incorporate independent readings from multiple observers to enable rigorous assessment of inter-observer variability. All ultrasound examinations and image interpretations were performed by experienced physicians from a high-volume tertiary centre. Diagnostic performance may not be reproduced in centres with less experienced operators. Nevertheless, we acknowledge an important limitation: DeLong test here only evaluates binary one-vs.-rest AUC comparison for each separate category; it does not provide an overall multi-class comparison across all ordered stages.

## Conclusions

Gastric filling ultrasonography provides detailed visualization of tumor morphology, lesional extent, depth of invasion, and perigastric lymph nodes. Its high sensitivity for early-stage disease positions it as a promising adjunctive screening tool, particularly for high-risk populations. As a non-invasive, low-cost, and radiation-free imaging option, ultrasound can be integrated into routine preoperative staging protocols to improve staging precision, its potential clinical utility for guiding surgical strategy remains to be confirmed by further prospective studies.

## Data Availability

The original contributions presented in the study are included in the article/Supplementary Material, further inquiries can be directed to the corresponding author/s.

## References

[1] Thrift AP Wenker TN El-Serag HB. Global burden of gastric cancer: epidemiological trends, risk factors, screening and prevention. Nat Rev Clin Oncol. (2023) 20(5):338–49. 10.1038/s41571-023-00747-0 36959359

[2] Pucułek M Machlowska J Wierzbicki R Baj J Maciejewski R Sitarz R. Helicobacter pylori associated factors in the development of gastric cancer with special reference to the early-onset subtype. Oncotarget. (2018) 9(57):31146–62. 10.18632/oncotarget.25757 PMC6089554 30123433

[3] Rawla P Barsouk A. Epidemiology of gastric cancer: global trends, risk factors and prevention. Prz Gastroenterol. (2019) 14(1):26–38. 10.5114/pg.2018.80001 30944675 PMC6444111

[4] Whiting J Sano T Saka M Fukagawa T Katai H Sasako M. Follow-up of gastric cancer: a review. Gastric Cancer. (2006) 9(2):74–81. 10.1007/s10120-006-0360-0 16767361

[5] In H Solsky I Palis B Langdon-Embry M Ajani J Sano T. Validation of the 8th edition of the AJCC TNM staging system for gastric cancer using the national cancer database. Ann Surg Oncol. (2017) 24(12):3683–91. 10.1245/s10434-017-6078-x 28895113

[6] Ye J Ren Y Wei Z Hou X Dai W Cai S. External validation of a modified 8th AJCC TNM system for advanced gastric cancer: long-term results in southern China. Surg Oncol. (2018) 27(2):146–53. 10.1016/j.suronc.2018.02.009 29937164

[7] Ungureanu BS Sacerdotianu VM Turcu-Stiolica A Cazacu IM Saftoiu A. Computed tomography for gastric cancer staging: a network meta-analysis. Diagnostics (Basel). (2021) 11(1):134. 10.3390/diagnostics11010134 33467164 PMC7829791

[8] Renzulli M Clemente A Spinelli D Ierardi AM Marasco G Farina D. Gastric cancer staging: is it time for magnetic resonance imaging? Cancers (Basel). (2020) 12(6):1402. 10.3390/cancers12061402 32485933 PMC7352169

[9] Sui A Hu Z Xie X Deng Y Wang Y Yu J. Automatic detection of gastric wall structure based on oral contrast-enhanced ultrasound and its application on tumor screening. Front Oncol. (2021) 11:627556. 10.3389/fonc.2021.627556 33854966 PMC8039386

[10] Wang L Liu Z Kou H He H Zheng B Zhou L. Double contrast-enhanced ultrasonography in preoperative T staging of gastric cancer: a comparison with endoscopic ultrasonography. Front Oncol. (2019) 9:66. 10.3389/fonc.2019.00066 30809510 PMC6380108

[11] Gao Z Lu Q Yan J Wang J Xu Z Jia J. The value of oral contrast-enhanced ultrasonography in the diagnosis of gastric tumors in the elderly. Panminerva Med. (2022) 64(4):576–7. 10.23736/S0031-0808.21.04466-9 34606184

[12] Wei F Huang P Li S Chen J Zhang Y Hong Y. Enhancement patterns of gastric carcinoma on contrast-enhanced ultrasonography: relationship with clinicopathological features. PLoS One. (2013) 8(9):e73050. 10.1371/journal.pone.0073050 24039857 PMC3765212

[13] Fukuya T Honda H Kaneko K Kuroiwa T Yoshimitsu K Irie H. Efficacy of helical CT in T-staging of gastric cancer. J Comput Assist Tomogr. (1997) 21(1):73–81. 10.1097/00004728-199701000-00014 9022773

[14] You M-W Park S Kang HJ Lee DH. Radiologic serosal invasion sign as a new criterion of T4a gastric cancer on computed tomography: diagnostic performance and prognostic significance in patients with advanced gastric cancer. Abdom Radiol. (2020) 45(10):2950–9. 10.1007/s00261-019-02156-3 31359095

[15] Harold KM MacCuaig WM Holter-Charkabarty J Williams K Hill K Arreola AX. Advances in imaging of inflammation, fibrosis, and cancer in the gastrointestinal tract. Int J Mol Sci. (2022) 23(24):16109. 10.3390/ijms232416109 36555749 PMC9781634

[16] Xu M Huang C-M Zheng C-H Li P Xie J-W Wang J-B. Does tumor size improve the accuracy of prognostic predictions in node-negative gastric cancer (pT1-4aN0M0 stage)? PLoS One. (2014) 9(7):e101061. 10.1371/journal.pone.0101061 25003849 PMC4086925

[17] Saito H Osaki T Murakami D Sakamoto T Kanaji S Oro S. Macroscopic tumor size as a simple prognostic indicator in patients with gastric cancer. Am J Surg. (2006) 192(3):296–300. 10.1016/j.amjsurg.2006.03.004 16920421

[18] Liu Z Ren W Guo J Zhao Y Sun S Li Y. Preliminary opinion on assessment categories of stomach ultrasound report and data system (Su-RADS). Gastric Cancer. (2018) 21(5):879–88. 10.1007/s10120-018-0798-x 29372460 PMC6097085

[19] Shen L Zhou C Liu L Zhang L Lu D Cai J. Application of oral contrast trans-abdominal ultrasonography for initial screening of gastric cancer in rural areas of China. Dig Liver Dis. (2017) 49(8):918–23. 10.1016/j.dld.2017.04.008 28487084

