# Supplementary material for: Preoperative staging of gastric cancer: a prospective comparison of gastric filling ultrasonography and enhanced CT with implications for surgical decision-making
Source: Front Surg. 2026 Oct 1;13:1925096. doi: 10.3389/fsurg.2026.1925096 (PMC13670109; doi:10.3389/fsurg.2026.1925096)
Supplement: Supplementary file 1 [file Table1.docx]

Supplement Table1：Comparison of the accuracy of gastric filling ultrasound and enhanced CT in determining T-staging

| Pathologic T-staging | n | Gastric filling ultrasound n（%） | Enhanced CT  n（%） | ***P*** |
| --- | --- | --- | --- | --- |
| T1 | 119 | 97（81.51） | 67（56.30） | 0.009 |
| T2 | 53 | 43（81.13） | 36（67.92） | <0.001 |
| T3 | 140 | 101（72.14） | 91（65.00） | 0.409 |
| T4 | 94 | 54（57.45） | 64（68.09） | 0.319 |
