# Supplementary material for: Preoperative staging of gastric cancer: a prospective comparison of gastric filling ultrasonography and enhanced CT with implications for surgical decision-making
Source: Front Surg. 2026 Oct 1;13:1925096. doi: 10.3389/fsurg.2026.1925096 (PMC13670109; doi:10.3389/fsurg.2026.1925096)
Supplement: Supplementary file 2 [file Table2.docx]

Supplement Table2：Comparison of the accuracy of gastric filling ultrasound and enhanced CT in determining N-staging

| Pathologic N-staging | n | Gastric filling ultrasound n（%） | Enhanced CT  n（%） | ***P*** |
| --- | --- | --- | --- | --- |
| N0 | 167 | 146（87.43） | 145（86.83） | 0.267 |
| N1 | 61 | 51（83.61） | 27（44.26） | 0.004 |
| N2 | 62 | 47（75.81） | 30（48.39） | 0.042 |
| N3 | 116 | 89（76.72） | 42（36.21） | <0.001 |
