# Supplementary material for: Preoperative staging of gastric cancer: a prospective comparison of gastric filling ultrasonography and enhanced CT with implications for surgical decision-making
Source: Front Surg. 2026 Oct 1;13:1925096. doi: 10.3389/fsurg.2026.1925096 (PMC13670109; doi:10.3389/fsurg.2026.1925096)
Supplement: Supplementary file 3 [file Table4.docx]

| Supplement Table 4.References for property values of clinical features. | |
| --- | --- |
| **Variables** | **Property Values** |
| **T stage** |  |
| T1 | 1 |
| T2 | 2 |
| T3 | 3 |
| T4 | 4 |
| T0 | 0 |
| **N stage** |  |
| N0 | 0 |
| N1 | 1 |
| N2 | 2 |
| N3 | 3 |
| **Sex** |  |
| Female | 0 |
| Male | 1 |
| **Age** |  |
| ≤55 | 0 |
| ＞55 | 1 |
| **Tumor location** |  |
| Cardia | 1 |
| Gastric fundus | 2 |
| Gastric antrum | 3 |
| Gastric angle | 4 |
| Gastric body | 5 |
| **Gross typing** |  |
| Early gastric cancer | 1 |
| Polyp type | 2 |
| Ulcer localized type | 3 |
| Ulcer infiltrating type | 4 |
| Diffuse infiltrating type | 5 |
| **Pathological types** |  |
| Adenocarcinoma | 1 |
| Neuroendocrine carcinoma | 2 |
| Mucinous adenocarcinoma | 3 |
| Adenosquamous carcinoma | 4 |
| Medullary carcinoma | 5 |
| **Lymph node metastasis** |  |
| No | 0 |
| Yes | 1 |
| **Abdominal wall thickness** |  |
| ≤2.5cm | 0 |
| ＞2.5cm | 1 |
| **Vascular infiltration** |  |
| No | 0 |
| Yes | 1 |
| **Perineural Invasion** |  |
| No | 0 |
| Yes | 1 |
| **Degree of differentiation** |  |
| Poorly differentiated | 1 |
| Moderately differentiated | 2 |
| Well-differentiated | 3 |
| Undifferentiated | 4 |
| **Tumor size** |  |
| ≤2.5cm | 0 |
| ＞2.5cm | 1 |
