# Supplementary material for: Preoperative staging of gastric cancer: a prospective comparison of gastric filling ultrasonography and enhanced CT with implications for surgical decision-making
Source: Front Surg. 2026 Oct 1;13:1925096. doi: 10.3389/fsurg.2026.1925096 (PMC13670109; doi:10.3389/fsurg.2026.1925096)
Supplement: Supplementary file 4 [file Table5.docx]

**Supplement Table5**: Univariate analysis logistic regression analysis of variables.

| Variable | OR | 95%CI‑lower | 95%CI‑upper | P‑value |
| --- | --- | --- | --- | --- |
| Vascular infiltration | 0.609 | 0.382 | 0.97 | 0.036 |
| Perineural Invasion | 0.582 | 0.338 | 1.001 | 0.044 |
| Gross typing | 0.821 | 0.66 | 1.022 | 0.037 |
| Lymph node metastasis | 0.735 | 0.468 | 1.154 | 0.181 |
| Degree of differentiation | 0.781 | 0.534 | 1.142 | 0.202 |
| Pathological types | 0.868 | 0.674 | 1.118 | 0.273 |
| Age | 0.758 | 0.425 | 1.352 | 0.348 |
| Tumor location | 1.078 | 0.902 | 1.288 | 0.412 |
| Tumor size | 0.81 | 0.472 | 1.388 | 0.443 |
| Pathologic N staging | 0.938 | 0.79 | 1.114 | 0.468 |
| Sex | 0.853 | 0.529 | 1.375 | 0.514 |
| Abdominal wall thickness | 0.964 | 0.539 | 1.725 | 0.903 |
