# Supplementary material for: Preoperative staging of gastric cancer: a prospective comparison of gastric filling ultrasonography and enhanced CT with implications for surgical decision-making
Source: Front Surg. 2026 Oct 1;13:1925096. doi: 10.3389/fsurg.2026.1925096 (PMC13670109; doi:10.3389/fsurg.2026.1925096)
Supplement: Supplementary file 5 [file Table6.docx]

**Supplement Table6**: Multivariate analysis logistic regression analysis of variables.

| Variable | OR | 95%CI‑lower | 95%CI‑upper | P‑value |
| --- | --- | --- | --- | --- |
| Vascular infiltration | 0.612 | 0.374 | 0.999 | 0.049 |
| Perineural Invasion | 0.691 | 0.401 | 1.191 | 0.185 |
| Gross typing | 0.868 | 0.701 | 1.075 | 0.193 |
