# Supplementary material for: Preoperative staging of gastric cancer: a prospective comparison of gastric filling ultrasonography and enhanced CT with implications for surgical decision-making
Source: Front Surg. 2026 Oct 1;13:1925096. doi: 10.3389/fsurg.2026.1925096 (PMC13670109; doi:10.3389/fsurg.2026.1925096)
Supplement: Supplementary file 6 [file Table7.docx]

Supplement Table7.STARD checklist

| Section and topic | Item No. | STARD 2015 Item text | Reported in manuscript (location) | Notes / Comments |
| --- | --- | --- | --- | --- |
| Title or abstract | 1 | Identify as a study of diagnostic accuracy using at least one measure of accuracy (such as sensitivity, specificity, predictive values, or AUC) | Abstract | Abstract mentions accuracy, AUC, κ‑statistics for T‑ and N‑staging |
|  | 2 | Structured summary of study design, methods, results and conclusions | Abstract | Structured abstract: Background‑Methods‑Results‑Conclusions |
| Introduction | 3 | Scientific and clinical background, including the intended use and clinical role of the index test | Introduction | Background of gastric cancer preoperative staging; role of gastric‑filling ultrasonography and enhanced‑CT |
|  | 4 | Study objectives and hypotheses | Introduction / Methods | Primary objective: compare diagnostic accuracy of two imaging modalities for preoperative gastric cancer T/N staging |
| Methods‑Study design | 5 | Whether data collection was planned before index‑test and reference‑standard performance (prospective) or after (retrospective) | Methods‑Study population | Prospective registered study |
| Methods‑Participants | 6 | Eligibility criteria (inclusion and exclusion criteria) | Methods‑Study population | Pre‑specified inclusion/exclusion criteria provided |
|  | 7 | Basis on which potentially eligible participants were identified | Methods‑Study population | Consecutive patients undergoing endoscopic biopsy‑proven gastric cancer |
|  | 8 | Setting and location where participants were identified, dates of enrolment | Methods‑Study population | Single tertiary hospital; Jan‑2022‑Jan‑2024 |
|  | 9 | Whether participants were consecutive, random, or convenience series | Methods‑Study population | Consecutive enrolment |
| Methods‑Test methods | 10a | Index test(s) described in sufficient detail to permit replication | Methods‑Scanning Methods | Gastric‑filling ultrasonography protocol |
|  | 10b | Reference standard described in sufficient detail to permit replication | Methods‑Staging criteria | Postoperative surgical histopathology (gold‑standard TNM staging) |
|  | 11 | Rationale for choice of reference standard | Methods‑Staging criteria | Postoperative pathology is accepted gold‑standard for gastric cancer TNM staging |
|  | 12a | Definition and rationale for index‑test positivity / result categories; distinguish pre‑specified vs exploratory cut‑offs | Methods‑Staging criteria; Methods‑Statistical analysis | Ultrasound / CT T‑, N‑staging criteria pre‑specified; abdominal‑wall‑thickness cut‑off 2.5 cm pre‑specified based on centre clinical experience; sensitivity analysis using continuous variable performed |
|  | 12b | Definition and rationale for reference‑standard result categories; distinguish pre‑specified vs exploratory cut‑offs | Methods‑Staging criteria | AJCC pathological TNM staging, pre‑specified |
|  | 13a | Whether readers of index test had access to clinical information and reference‑standard results | Methods‑Image interpretation | Blinded: ultrasound readers blinded to CT results; discrepancies resolved by consensus |
|  | 13b | Whether assessors of reference standard had access to index‑test results | Methods‑Image interpretation | Pathology reviewers blinded to imaging findings |
| Methods‑Statistical methods | 14 | Methods used to estimate / compare measures of diagnostic accuracy | Methods‑Statistical analysis | McNemar test, DeLong test for AUC comparison, Kappa, logistic regression; 95 %CI reported |
|  | 15 | How indeterminate / non‑evaluable index‑test results were handled | Methods‑Study population; Figure 1 | Non‑evaluable ultrasound cases excluded before final analysis, shown in participant flowchart |
|  | 16 | How missing data for index‑test / reference‑standard were handled | Methods‑Statistical analysis; Table footnotes | Zero missing data among final analysed cohort n=406; no imputation performed |
|  | 17 | Any analyses of variability / subgroup analyses; distinguish pre‑specified vs exploratory | Results; Supplementary tables | Subgroup accuracy comparisons; exploratory logistic‑regression and stratified prediction model (secondary exploratory analysis) |
|  | 18 | Intended sample size and how it was determined | Methods‑Statistical analysis | Final enrolled n=406 |
| Results‑Participants | 19 | Participant flow (STARD‑recommended flowchart) | Figure 1 | Flowchart showing screening, exclusions, non‑evaluable cases, final analysed n=406 |
|  | 20 | Baseline demographic and clinical characteristics of study participants | Table 1, Table 2 | Age, sex, tumour location, gross type, differentiation etc. |
|  | 21a | Distribution of target‑condition severity among participants with target condition | Table 1‑6 | Pathological T‑stage: T1 n=119; T2 n=53; T3 n=140; T4 n=94; N‑stage distribution also provided |
|  | 21b | Distribution of alternative diagnoses among participants without target condition | Not applicable | All participants have histologically confirmed gastric cancer |
|  | 22 | Time interval between index‑test(s) and reference‑standard, and any intervening clinical interventions | Methods‑Scanning Methods | Both index tests performed ≤7 days before surgical resection; no intervening neoadjuvant therapy in analysed cohort |
| Results‑Test results | 23 | Cross‑tabulation of index‑test results against reference‑standard results | Table 3‑6 | Full contingency / cross‑tabulation for ultrasound‑T, CT‑T, ultrasound‑N, CT‑N vs pathological gold‑standard |
|  | 24 | Estimates of diagnostic accuracy together with precision (e.g. 95 % confidence intervals) | Main‑text, Tables, Supplementary tables | Accuracy, sensitivity, specificity, AUC, Kappa, OR all reported with 95 %CI |
|  | 25 | Any adverse events related to index‑test or reference‑standard procedures | Methods‑Scanning Methods | No adverse events from oral ultrasound contrast or intravenous CT contrast agent |
| Discussion | 26 | Study limitations including potential bias, statistical uncertainty, generalisability | Discussion‑Limitations | Single‑centre; only resected patients; no T1a/T1b separation; no M‑stage / peritoneal assessment; exploratory model not externally validated |
|  | 27 | Implications for practice, intended clinical role of index‑test | Discussion | Emphasis: diagnostic‑performance results only; proposed workflow framed as hypothesis for future validation, not established clinical algorithm |
| Other information | 28 | Study registration identifier and register name | Abstract, Methods | ClinicalTrials.gov NCT05596864 |
|  | 29 | Where the full study protocol can be accessed | Methods | Protocol publicly available via ClinicalTrials.gov website |
|  | 30 | Sources of funding and other support; role of funders | Manuscript footnote / Acknowledgements | Jilin Province Science and Technology Development Plan grant; funder had no role in study design, analysis or manuscript writing |
